# *Mikania glomerata*: Phytochemical, Pharmacological, and Neurochemical Study

**DOI:** 10.1155/2014/710410

**Published:** 2014-08-18

**Authors:** Lorena C. L. R. Santana, Maria R. M. Brito, George L. S. Oliveira, Antônia M. G. L. Citó, Clayton Q. Alves, Juceni P. David, Jorge M. David, Rivelilson M. de Freitas

**Affiliations:** ^1^Departamento de Farmácia, Laboratório de Pesquisa em Neuroquímica Experimental do Programa de Pós-Graduação em Ciências Farmacêuticas, Universidade Federal do Piauí, 64049-550 Teresina, PI, Brazil; ^2^Programa de Pós-Graduação em Biotecnologia (RENORBIO), Universidade Federal do Piauí, 64049-550 Teresina, PI, Brazil; ^3^Departamento de Química, Centro de Ciências da Natureza, Universidade Federal do Piauí, Campus Universitário Ministro Petrônio Portella, Bairro Ininga, 64049-550 Teresina, PI, Brazil; ^4^Departamento de Ciências Exatas, Universidade Estadual de Feira de Santana, 44031-460 Feira de Santana, BA, Brazil; ^5^Faculdade de Farmácia, Universidade Federal da Bahia, 40170-290 Salvador, BA, Brazil; ^6^Instituto de Química, Universidade Federal da Bahia, 40170-290 Salvador, BA, Brazil

## Abstract

The present study primarily aims to identify the relative density and the fatty acids (methyl esters) content present in the standardized ethanol extract of leaves of *M. glomerata* (EPMG). Meanwhile, in a second moment, this study evaluated the effects of the EPMG on the levels of amino acids in the hippocampus, and the mechanism of sedative and anxiolytic action. Adult mice were treated with doses of 200, 300, and 400 mg/kg and evaluated in open field, elevated plus-maze, light dark, and rotarod tests. Moreover, in the behavioral tests diazepam (GABAergic anxiolytic, 2 mg/kg) as positive control and flumazenil (GABA antagonist, 2.5 mg/kg) were used to identify mechanism of sedative and anxiolytic action produced by EPMG. The EPMG is constituted by the following compounds: methyl cinnamate, 2H-1-benzopyran-2-one, (2-hydroxyphenyl)methyl propionate, (Z)-methyl-hexadec-7-enoate, methyl hexadecanoate, hexadecanoic acid, (Z)-methyl-octadec-9-enoate, octadecanoic acid, and squalene. This extract demonstrated anxiolytic effects, which may be mediated by GABAergic system, and was able to increase GABA levels and reduce of glutamate and aspartate concentrations in mice hippocampus, which can directly and/or indirectly assist in their anxiolytic effect. Although more studies are needed, the EPMG could represent an interesting therapeutical strategy in the treatment of anxiety.

## 1. Introduction

*Mikania glomerata* Sprengel (*M. glomerata*, Asteraceae) is a species belonging to the genus* Mikania* (tribo Eupatorieae, subtribo Miikaniinae) and popularly known as “guaco,” which became official in the first Brazilian Pharmacopoeia because of its therapeutic properties as bronchodilator, antiallergic, and antiasthmatic [1–3]. In addition to the popular use, the* M. glomerata* presents other therapeutic properties such as antihemorrhagic [4], antiophidian [5], antiviral [6], and antimicrobial [7].

Phytochemical studies identified in the genus* Mikania* the presence of coumarin, diterpenes, lactones, and sesquiterpenes [8, 9]. The constituents of the species* M. glomerata* were identified as coumarin, lupeol acetate* o*-hydroxy-cinnamic acid [10], kaurenoic acid [11], cinnamoylgrandifloric acid, and stigmasterol [12]. Most of these compounds were proven to have pharmacological activity: lupeol has anti-inflammatory activity [13]; kaurenoic acid is a potential antimicrobial, hypotensive, and anti-inflammatory; coumarin is an anti-inflammatory, immunosuppressive, antihypertensive, and antioxidant [3]; and stigmasterol has antinociceptive, anti-inflammatory, and hypocholesterolemic activity [14].

In spite of its pharmacological properties previously discussed, studies with implications on the central nervous system for* M. glomerata* are quite scarce in the scientific literature, with only studies for different species of the same genus being found [15], which reinforces the need for further studies for this plant species. Thus, the present study aims to determine neuropharmacological activity of the standardized ethanol extract of leaves of* M. glomerata* (EPMG) by evaluation of anxiolytic effect and their possible action mechanism in animal models. The amino acids levels (*γ*-aminobutyric acid (GABA), glutamate, glutamine, and aspartate) in mice hippocampus, antioxidant capacity, and chemical constituents of fatty acids were also determined. So, the evaluation of neuropharmacological effects of* M. glomerata* will be important to establish a new pharmacological strategy as medicinal herb.

## 2. Material and Methods

### 2.1. Reagents

Diazepam (DZP) 2 mg/kg (Sigma Chem. Co., St. Louis, MO, USA) and flumazenil (FLU) 2.5 mg/kg (Sigma Chem. Co., St. Louis, MO, USA) were used as standards. 2,2-Diphenyl-1-picrylhydrazyl (DPPH) radical, 2,2′-azinobis(3-ethylbenzthiazoline-6-sulfonic acid) (ABTS), and Trolox (Sigma Chem. Co., St. Louis, MO, USA) were used to evaluate the antioxidant capacity. All chemicals used were of highest available purity grade.

### 2.2. Vegetable Material and Collecting the Leaves from* M. glomerata*

The aerial parts of* M. glomerata* were collected in the Garden of Medicinal Plants of Phytotherapy Center (NUFITO), which is part of Center for Pharmaceutical Care (NUASF) of the Health Secretariat of the State of Ceará (SESA). A voucher specimen (#27,041) was deposited in the Herbarium Graziella Barroso at Federal University of Piaui. The botanical material was collected manually and then rinsed in tap water, followed by distilled water. After the search for foreign materials, the plant material was dried in the shade.

### 2.3. Preparation of Standardized Ethanol Extract of Leaves of* M. glomerata*

According to the method described by Santana et al. [16], the extract was prepared by percolation with previous soaking for 24 h, using 70% hydroethanolic solution, with the ratio of 15 mL solution per 1 g of vegetable drug. The extraction solution was then concentrated in an oven with forced air circulation to eliminate the ethanol content and increase the solids content, which corresponded to a reduction of 75% of the initial volume. After vacuum filtration, the filtrate was concentrated using a rotary evaporator and then lyophilized.

### 2.4. Determination of Relative Density of Standardized Ethanol Extract of Leaves from* M. glomerata*

The determination of the relative density of the extract was performed using the pycnometer according to the Brazilian Pharmacopoeia in duplicate [17].

### 2.5. Chemical Profile of Methyl Esters Present in Standardized Ethanol Extract of Leaves of* M. glomerata*

The fatty acid methyl esters were obtained by the method described by Hartman [18] with modifications. A sample of 500 mg of standardized ethanolic extract of leaves of* M. glomerata* was added to a 50 mL flask to which 5 mL of NaOH 0.5 mol/L dissolved in methanol was added. The mixture was refluxed for 5 minutes and added to 15 mL of esterifying reagent (a mixture prepared at reflux for 15 minutes, 2 g of NH_4_Cl, 60 mL of MeOH, and 3 mL of concentrated H_2_SO_4_). The esterification reaction was held at reflux for 10 minutes and transferred to a separatory funnel, followed by addition of 25 mL of hexane and 50 mL of distilled water. After careful stirring and phase separation, the aqueous phase was discarded and the hexane phase analyzed by injection into a gas chromatograph coupled with a mass spectrometer (GC/MS).

### 2.6. HPLC Profile and Chemical Marks of* M. glomerata* Ethanolic Extract

The HPLC profile of the ethanolic extract of* M. glomerata* was obtained in a Dionex DAD liquid chromatograph model UltiMate 3000. The chromatograms were registered employing a 5 *μ*m, 2.1 × 100 mm RP-18 column (Dionex Acclaim), the oven temperature was set at 30°C, and the injection volume was set as 10 *μ*L in the autosampler and *λ* = 330 nm. The best conditions for the mobile phase were a 0–100% gradient of MeOH : H_2_O (0.1% acetic acid) and a flow rate of 0.8 mL/min. The MeOH (Tedia, Fairfield, USA) and acetic acid (Merck, Darmstadt, Germany) used were of HPLC grade. Water was supplied by a Milli-Q water purifier system from Millipore (Bedford, MA, USA) and was used after filtration through a 0.2 *μ*m pore size membrane filter. Coumarin and 2-hydroxycinnamic acid standards were obtained from Sigma (St. Louis, MO, USA). This analytical method described has been developed by our research group for the detection and quantification of their main constituents and involved the optimization of several stages such as the preparation of the sample, chromatographic separation, and quantification. The validation of the analytical method was performed to ensure the success of the use of our methodology, in addition to detecting errors of analytical procedure, and provide proven evidence of efficiency of the method.

### 2.7. Antioxidant Potential of Standardized Ethanolic Extract of Leaves of* M. glomerata*

The antioxidant study was determined by scavenging the stable free radical DPPH^•^ (1,1-diphenyl-2-picrylhydrazyl) and ABTS^•+^ (2,2′-azinobis-3-ethylbenzothiazoline-6-sulfonic acid) by compounds present in standardized ethanolic extract of leaves of* M. glomerata*.

For the DPPH^•^ test, the methodology described by Silva et al. [19] was used with minor modifications. A stock solution of the standardized ethanol extract of leaves of* M. glomerata* (150 mg/mL), of DPPH^•^ (40 *μ*g/mL), and of standard Trolox (94 *μ*g/mL) was prepared. The concentrations used were 12.5, 25, 50, 75, and 100 *μ*g/mL, all being prepared by dilution from the highest concentration. The reaction mixture (0.3 mL of extract plus 2.7 mL of a stock solution of DPPH^•^) was incubated at room temperature in the absence of light (in the dark) for 30 minutes and the absorbance was measured in a spectrophotometer at 517 nm. The antioxidant evaluation was performed in triplicate and the absorbance values were expressed as percentage of inhibition of absorbance of DPPH^•^ solution by the following equation: % of inhibition of radical DPPH^•^ = {(*A*_control_ − *A*_reaction  mixture_) × 100}/*A*_control_ in which *A*_control_ is the initial absorbance of the ethanol solution of DPPH^•^ and *A*_reaction  mixture_ is the absorbance of the reaction mixture containing the DPPH^•^ and the concentrations of ethanol extract under study. The inhibitory concentration (IC_50_) of the standardized ethanol extract of leaves of* M. glomerata* required to reduce the absorbance of DPPH^•^ to 50% at 517 nm was determined.

For determination of antioxidant capacity by method of ABTS^•+^ the method described by Re et al. [20] was used with modifications. Initially the radical cation ABTS^•+^ from the reaction of 5 mL of a 7 mM ABTS^•+^ with 88 *μ*L of a solution 2.45 mM potassium persulphate (K_2_S_2_O_8_) was formed and incubated at room temperature in the absence of light for 16 hours. Elapsed this time, a solution of ABTS^•+^ was diluted in ethanol to obtain a solution with absorbance of 0.70 (±0.05), at 734 nm. Final concentrations used were 12.5, 25, 50, 75, and 100 *μ*g/mL of the standardized ethanolic extract of leaves of* M. glomerata*. In the dark, at room temperature, an aliquot of 40 mL of each concentration was transferred to test tubes with 1960 *μ*L of radical ABTS^•+^.

The reading of absorbance was performed at room temperature, at time of 6 minutes in a spectrophotometer, at 734 nm, and the results were expressed as percentage inhibition of the absorbance of the solution ABTS^•+^ by the following equation: % of inhibition of radical ABTS^•+^ = {(*A*_control_ − *A*_reaction  mixture_) × 100}/*A*_control_, in which *A*_control_ is the initial absorbance of the ethanolic solution of ABTS^•+^ and *A*_reaction  mixture_ is the absorbance of the reaction mixture containing ABTS^•+^ and at concentrations of ethanolic extract under study. The inhibitory concentration (IC_50_) of the standardized ethanol extract of leaves of* M. glomerata* required to reduce the absorbance of ABTS^•+^ to 50% at 734 nm was determined.

### 2.8. Animals and Experimental Protocols

Adult male (2 months old) mice of the albino* Swiss* strain, weighing 25–30 g, were provided by Central Animal Facility of Center for Agricultural Sciences, Federal University of Piaui (UFPI). During all experiments, the animals were acclimated to 26 ± 1°C and kept in acrylic cages of 30 × 30 cm^2^ with a maximum of six animals for observation of parameters related to acute toxicity initially during the first 24 h of the study. The animals were kept in similar environmental conditions, with light/dark cycle of alternating 12 h, being fed at a basal type Purina diet and water* ad libitum*. These experiments were approved by Ethic Committee in Animal Experimentation of Federal University of Piaui (#0071/10).

The lyophilized residue from the standardized ethanol extract of* M. glomerata* was diluted in distilled water to obtain a final concentration of 10, 20, and 40 mg/mL to carry out the treatment of animals.

To evaluate the possible sedative, anxiolytic, and muscle relaxant effects, the animals were divided into six groups of twelve animals and were subsequently treated for a period of 30 consecutive days with 0.25 mL of 0.9% saline (vehicle-control, p.o.), diazepam 2 mg/kg (positive control-DZP 2, i.p.), flumazenil at a dose of 2.5 mg/kg (group FLU 2.5, i.p.), and standardized ethanolic extract of leaves of* M. glomerata* at doses 200, 300, and 400 mg/kg (p.o.) corresponding to groups EPMG 200, EPMG 300, and EPMG 400, respectively. The doses used were based on a preliminary pharmacological screening protocol [21] with different doses of the standardized ethanol extract of* M. glomerata* administered to mice. At all doses used, no signs of acute toxicity or behaviors suggestive of neurotoxicity were observed (data not shown).

To clarify the mechanism of action, four groups of twelve animals were treated for a period of 30 consecutive days with flumazenil at a dose of 2.5 mg/kg (i.p.) and 30 min after the last dose of the treatment were administered with diazepam 2 mg/kg (FLU 2.5 + DZP 2) and standardized ethanol extract of leaves of* M. glomerata* at doses of 200, 300, and 400 mg/kg corresponding to groups FLU 2.5 + EPMG 200, FLU 2.5 + EPMG 300, and FLU 2.5 + EPMG 400, respectively. After 30 minutes of the last administration, the animals were evaluated in experimental protocols described below.

### 2.9. Evaluation of Locomotor Activity and Motor Coordination in Mice Treated with Standardized Ethanol Extract of Leaves of* M. glomerata*

The motor activity of the animals was checked by means of an open field with dimensions of 30 × 30 × 15 cm, made of transparent acrylic walls, black floor and divided into 09 equal quadrants [22]. After 30 minutes of administration of the last dose related to treatment of animals, one at a time was placed in the center of the field where the number of intersections with four legs, the number of self-grooming behaviors (grooming), and the number of vertical explorations (rearing), not lean against the wall, were recorded during the time of 5 minutes. After each test, the equipment was cleaned with a solution of 70% ethanol. After this procedure the device was again cleaned with a sponge soaked in water.

The effect of muscle relaxant and motor coordination of the animals treated as experimental protocol was observed in the rotarod test [23]. For this test, 30 minutes after the last dose for the treatment of animals, one at a time was placed on four legs with a bar of 2.5 cm in diameter, 25 cm high from the floor in a rotation of 16 rpm for a period of 3 minutes. In this test, residence time in the rotating bar, in seconds, and the number of falls, with three renewals maximum, were recorded. After each test, the equipment was cleaned with a solution of 70% ethanol. After this procedure the device was again cleaned with a sponge soaked in water.

### 2.10. Evaluation of Anxiolytic Activity in Mice Treated with Standardized Ethanol Extract of Leaves of* M. glomerata*

In evaluating the anxiolytic activity, the elevated plus maze test (EPM) was used, consisting of two open and opposed arms, and two other opposed closed arms of equal size (50 × 10 cm each), made of wood, with side walls measuring 50 cm high. At the edges of the open arms, a small wooden ledge (0.5 cm) is fixed in order to reduce the number of falls of the animals. The arms perpendicularly intersect to form a cross, bounded by a central 10 × 10 cm area and 50 cm high from the ground. The maze is in a soundproof room, partially illuminated by an incandescent lamp (60 W), and is placed vertically, 150 cm above the apparatus [24].

In this experiment, 30 minutes after the last dose, each animal was placed in the center of the maze facing one of the closed arms. We recorded the number of entries and time spent in the open arms for a period of five minutes, recording the number of entries (NEOA) and total time spent in open arms (TTOA) in seconds. After each test, the equipment was cleaned with a solution of 70% ethanol. After this procedure the device was cleaned again with a sponge soaked in water.

Complementing these data in the light/dark box test, 30 minutes after the last dose, each mouse was placed in the center of the illuminated part facing the opening that leads to the dark side of the box, one at a time; the equipment is described below. The apparatus used is made of acrylic and is divided into two compartments (light compartment and dark compartment) that communicate [25]. The dark compartment (black acrylic, 27 × 18 × 29 cm) is poorly lit. The light compartment (transparent acrylic, 27 × 18 × 29 cm) in all tests was directly illuminated by a fluorescent lamp (cold) of 20 W light power, while the dark part was fitted with a black cap. Each animal was intensively observed and, for five minutes after the first entry in the dark side, we recorded behavioral parameters and after each test the equipment was cleaned with a solution of 70% ethanol. After this procedure, the device was again cleaned with a sponge soaked in water. The parameter used in this test was the time spent in the light compartment and is expressed in seconds.

### 2.11. Evaluation of Amino Acids Levels in Mice Hippocampus Treated with Standardized Ethanol Extract of Leaves of* M. glomerata*

To evaluate the amino acid levels (GABA, glutamate, glutamine, and aspartate) the animals were divided into four groups of ten, and they were subsequently treated for a period of 30 consecutive days with 0.25 mL saline 0.9% (vehicle-control, p.o.) and standardized ethanol extract of leaves of* M. glomerata* at doses 200, 300, and 400 mg/kg corresponding to groups EPMG 200, EPMG 300, and EPMG 400, respectively.

After behavioral testing the animals were sacrificed by administering sodium pentobarbital (40 mg/kg, i.p.). Then, their brains were dissected on ice to remove the hippocampus on both sides to determine amino acid levels (GABA, glutamate, aspartate, and glutamine). The hippocampus was used to prepare 10% homogenates. The brain tissues were sonicated in perchloric acid (HClO_4_) for 30 seconds and centrifuged for 15 minutes in a refrigerated centrifuge at 21500 ×g. The supernatant was separated and filtered through a membrane filter (Millipore 0.2 mM) and then combined with a solution of precolumn derivatization to obtain fluorescence in a ratio of 1 : 1. One minute after the start of this association, an aliquot of 20 *μ*L was removed and injected into the HPLC equipment for chemical analysis.

For amino acid analysis, a CLC-ODS column (M), with a length of 15 cm in diameter, 4.6 mm gauge, and particle diameter of 3 *μ*m, (Shimadzu, Japan) was used. The mobile phase used was in gradient using two phases: (A) NaH_2_PO_4_ (50 mM) and methanol (20% v/v) at pH 5.5 and (B) pure methanol (100%). Aspartate (ASP), glutamate (Glu), glutamine (GLN), and *γ*-aminobutyric acid (GABA) were detected using a fluorescence detector (Model RF-535 from Shimadzu, Japan) with wavelengths EX-wavelength (370 nm) and MS-wavelength (450 nm). The chromatograms were recorded and quantified by a computer using software from Shimadzu. The amount of amino acid was calculated by comparing the peak height obtained from the average of the patterns, and the results were expressed in *μ*mol/g of tissue.

### 2.12. Statistical Analysis

Results were expressed as the mean ± the standard error of the mean (SEM) of number of animals that were used in the experiments. The values that followed a parametric distribution were analyzed by the analysis of variance (ANOVA) and the *t*-Student-Newman-Keuls test as* post hoc* test, using the program GraphPad Prism version 5.00 for Windows. Differences were considered statistically significant from *P* < 0.05.

## 3. Results

### 3.1. Relative Density and Chemical Profile

The relative density found for the EPMG was 1.0142 g/cm^3^, indicating that this extract is slightly denser than water. Fatty acids such as omega-3, particularly eicosapentaenoic acid (EPA) and docosahexanoic (DHA), develop an important role in the central nervous system. However, for the fatty acids identified as methyl esters in the extract of* M. glomerata* (Table 1) reports in the literature on their activity in central nervous system were not found.

**Figure 1:**
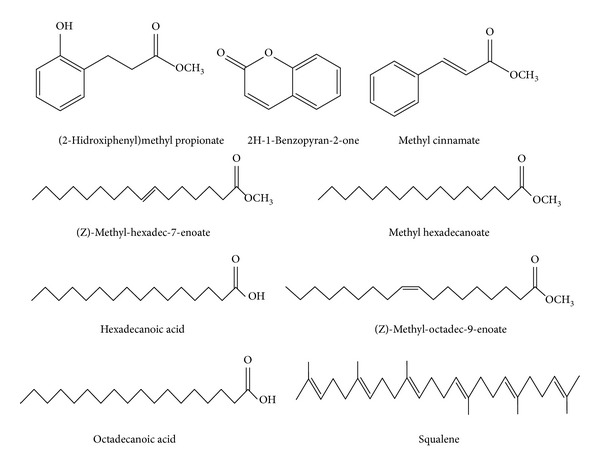
Chemical structure of fatty acids methyl esters and other constituents identified by GC/MS after hydrolysis and methylation of standardized ethanol extract of leaves of* M. glomerata* Sprengel.

**Figure 2:**
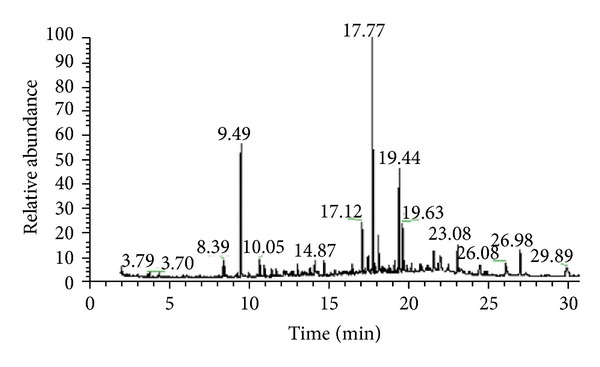
Chromatogram of standardized ethanol extract of leaves from* M. glomerata* Sprengel for identification of fatty acid methyl esters and other constituents by GC/MS, after hydrolysis and methylation.

The main chemical constituents (Figure 1) identified in chromatogram (Figure 2) of the EPMG were methyl cinnamate, 2H-1-benzopyran-2-one, (2-hydroxyphenyl)methyl propionate, (Z)-methyl-hexadec-7-enoate, methyl hexadecanoate, hexadecanoic acid, (Z)-methyl-octadec-9-enoate, octadecanoic acid, and squalene (Table 1).

**Figure 3:**
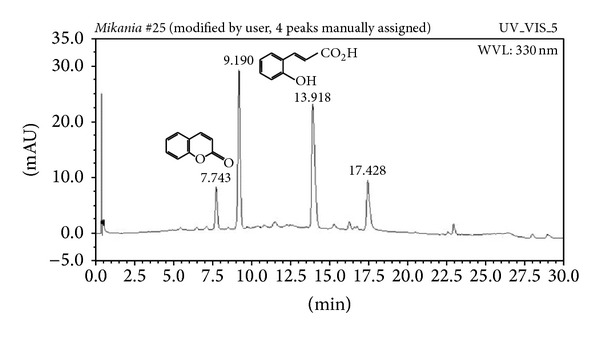
HPLC profile of the standardized ethanol extract of leaves of* M. glomerata* Sprengel.

The HPLC/DAD profile of EPMG indicates very few phenolic compounds present in this extract (Figure 3). Coumarin and 2-hydroxycinnamic acids are present and these compounds are well established in this species. So, they could be considered chemical markers of standardized extracts of this plant once they present specific ultraviolet spectra and are easily separated by this technique. Quantitative analyses of both compounds in this extract indicate the presence of 1.34 ± 0.014% of 2-hydroxycinnamic acid and 0.151 ± 0.001% of coumarin in samples analysed.

### 3.2. Antioxidant Potential* In Vitro*

**Figure 4:**
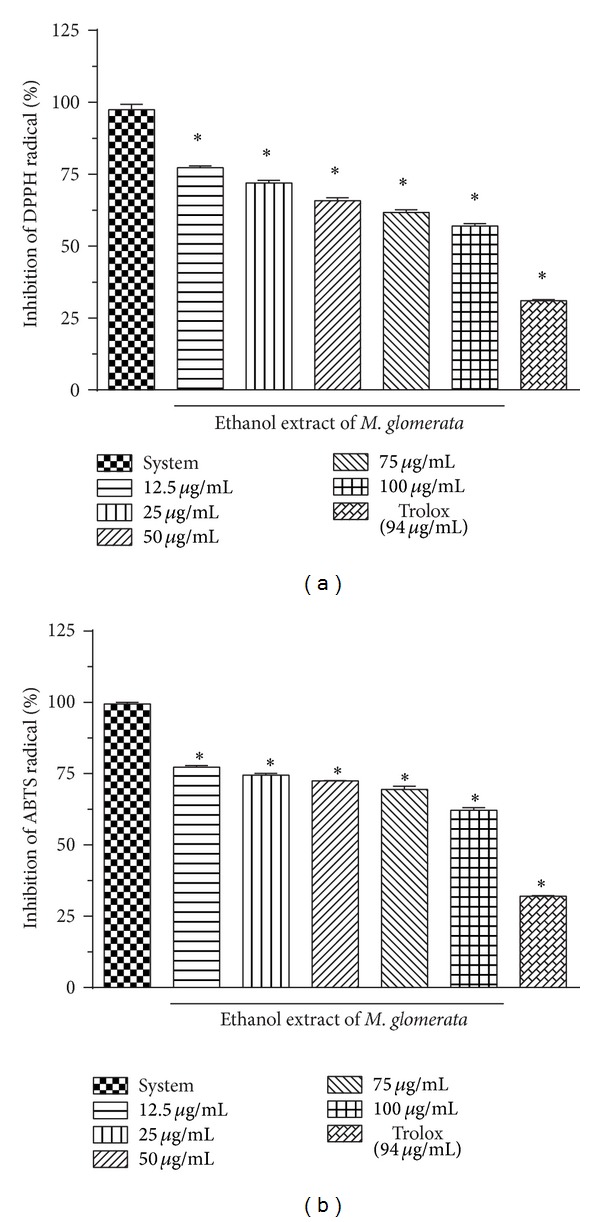
Antioxidant capacity of standardized ethanol extract of leaves from* M. glomerata* by inhibition of DPPH^•^ and ABTS^•+^ radicals. Values represent the mean ± SEM (*n* = 3). *P < 0.05 compared with system (ethanolic solution of DPPH^•^ and ABTS^•+^).

In order to evaluate the* in vitro* antioxidant capacity of EPMG, the use of DPPH^•^ and ABTS^•+^ radical was considered as a simple and rapid procedure. The results for the antioxidant capacity at different concentrations are shown in Figure 4. The values of the antioxidant activity against DPPH^•^ radical at concentrations of 12.5, 25, 50, 75, and 100 *μ*g/mL were 22.73 ± 0.64, 28.06 ± 0.90, 34.16 ± 0.94, 38.3 ± 0.96, and 43.03 ± 0.81%, which decreased significantly (*P* < 0.05) the concentration of the solution of DPPH^•^. The Trolox (94 *μ*g/mL) also reduced the DPPH^•^ radical presenting 69 ± 0.43% of antioxidant capacity. As for the DPPH^•^ radical, a similar significant reduction (*P* < 0.05) was also observed for the ABTS^•+^ radical by the extract at study that showed an antioxidant capacity of 21.56 ± 0.20, 25.56 ± 0.72, 27.46 ± 0.05, 30.5 ± 1.05, and 37.83 ± 0.96% for concentrations of 12.5, 25, 50, 75, and 100 *μ*g/mL, respectively. The Trolox showed 67.9 ± 0.17% of antioxidant capacity, and this result was greater than the antioxidant capacity presented by samples by EPMG in concentration of 100 *μ*g/ml. According to the results of antioxidant capacity, the inhibitory concentration (IC_50_) of EPMG required to reduce DPPH^•^ and ABTS^•+^ at 50% of its initial concentration was 138.91 *μ*g/mL and 175.68 *μ*g/mL, respectively.

### 3.3. Evaluation of Locomotor Activity and Motor Coordination

In the open field test, the groups treated with EPMG at the three doses did not present alterations in the number of crossings (*F*_10_ = 1370, *r* = 0.99, *P* > 0.001) and rearings (*F*_10_ = 799.5, *r* = 0.99, *P* > 0.001), but it had changes in the number of groomings (*F*_10_ = 21.99, *r* = 0.66, *P* > 0.01). However, the group treated with DZP reduced the number of crossings (*F*_10_ = 1370, *r* = 0.99, *P* > 0.001), rearings (*F*_10_ = 799.5, *r* = 0.99, *P* > 0.001), and groomings (*F*_10_ = 21.99, *r* = 0.66, *P* > 0.01), when compared to the group treated with vehicle (negative control). On the other hand, the group treated with the three doses EPMG only increased the number of crossings (*F*_10_ = 1370, *r* = 0.99, *P* > 0.001) and rearings (*F*_10_ = 799.5, *r* = 0.99, *P* > 0.001), compared to DZP-treated group, suggesting that the sedative effect of the extract is lower than that produced by diazepam, since only one of the parameters has changed (groomings).

In the open field test, flumazenil reversed the effect of diazepam on the number of crossings (*F*_10_ = 1370, *r* = 0.99, *P* > 0.001), groomings (*F*_10_ = 21.99, *r* = 0.66, *P* > 0.01), and rearings (*F*_10_ = 799.5, *r* = 0.99, *P* > 0.001), when compared to the group treated only with DZP (Table 2).

In the same test, flumazenil, in turn, did not reverse the effect of the EPMG on the number of intersections at doses of 400 mg/kg (*F*_10_ = 1370, *r* = 0.99, *P* > 0.001), 300 mg/kg (*F*_10_ = 1370, *r* = 0.99, *P* < 0.001), and 200 mg/kg (*F*_10_ = 1370, *r* = 0.99, *P* > 0.001). In relation to the number of groomings (*F*_10_ = 21.99, *r* = 0.66, *P* > 0.05) and rearings (*F*_10_ = 799.5, *r* = 0.99, *P* > 0.001), no change was observed, suggesting that the EPMG does not act by GABA_A_ receptor (Table 2).

### 3.4. Evaluation of Anxiolytic Activity

In the light/dark box test, the EPMG at the dose of 400 mg/kg increased the time spent in the light compartment of the treated animals when compared to the negative control (*F*_10_ = 553, *r* = 0.99, *P* > 0.001) and the diazepam group (200 mg/kg (*F*_10_ = 553, *r* = 0.99, *P* > 0.001), 300 mg/kg (*F*_10_ = 553, *r* = 0.99, *P* > 0.001), and 400 mg/kg (*F*_10_ = 553, *r* = 0.99; *P* > 0.001)) (Table 3).

The animals treated with doses of 200 mg/kg (*F*_10_ = 553, *r* = 0.99, *P* > 0.001), 300 mg/kg (*F*_10_ = 553, *r* = 0.99, *P* > 0.001), and 400 mg/kg (*F*_10_ = 553, *r* = 0.99, *P* > 0.001) produced an increase in this parameter only when compared to the vehicle group (negative control), suggesting that this extract has anxiolytic effects at the doses tested (Table 3).

In the same test, it was found that flumazenil reversed the effect on time spent in the light compartment at doses of 200 mg/kg (*F*_10_ = 553, *r* = 0.99, *P* > 0.001), 300 mg/kg (*F*_10_ = 553, *r* = 0.99, *P* < 0.001), and 400 mg/kg (*F*_10_ = 553, *r* = 0.99, *P* > 0.001) of the EPMG. These results suggest that the anxiolytic effect can be mediated by GABA_A_ receptors. Likewise, flumazenil could reverse the DZP effect (*F*_10_ = 553, *r* = 0.99, *P* > 0.001).

In rotarod test, the EPMG increased the number of falls (*F*_10_ = 3.31, *r* = 0.23, *P* > 0.05) and reduced the time spent on the rotating bar (*F*_10_ = 6.91, *r* = 0.39, *P* > 0.001), only at the dose of 400 mg/kg when compared to the negative control group. The doses, in turn, produced no muscle relaxant effect superior to DZP (*P* > 0.05). However, the effects on motor coordination of group DZP (number of falls (*F*_10_ = 3.31, *r* = 0.23, *P* > 0.01) and time spent on the rotating bar (*F*_10_ = 6.91, *r* = 0.39, *P* < 0.001)) and the dose of 400 mg/kg of the EPMG (number of falls (*F*_10_ = 3.31, *r* = 0.23, *P* > 0.05) and time spent on the rotating bar (*F*_10_ = 6.91, *r* = 0.39, *P* > 0.05)) were reversed by flumazenil, suggesting that the EPMG produces incoordination, and only at the highest dose similar to DZP by GABA receptors (Table 4).

In the rotarod test flumazenil did not alter the effect of the EPMG on motor coordination of the mice in the number of falls at doses of 200 mg/kg (*F*_10_ = 3.31, *r* = 0.23, *P* > 0.05) and 300 mg/kg (*F*_10_ = 3.31, *r* = 0.23, *P* > 0.05, Table 4). Additionally, it produced no change on the residence time on the rotating bar, in the animals treated with doses of 200 mg/kg (*F*_10_ = 6.91, *r* = 0.39, *P* > 0.05) and 300 mg/kg (*F*_10_ = 6.91, *r* = 0.39, *P* > 0.05), when compared to the groups treated with EPMG 400 mg/kg (Table 4).

In EPM test, we found that the EPMG, only at the highest dose, increased the parameter values NEOA (*F*_10_ = 18.51, *r* = 0.63, *P* > 0.001) and TTOA EPMG 400 mg/kg (*F*_10_ = 1442, *r* = 1.0, *P* < 0.001), compared to negative control. Likewise, only that dose produced an increase in NEOA (*F*_10_ = 18.51, *r* = 0.63, *P* > 0.05) and TTOA (*F*_10_ = 1442, *r* = 1.0, *P* < 0.001) when compared to DZP group. In the group treated with DZP, an increase in NEOA (*F*_10_ = 18.51, *r* = 0.63, *P* > 0.001) and TTOA (*F*_10_ = 1442, *r* = 1.0, *P* < 0.001) was also observed, when compared with negative control (Table 5).

In this test, flumazenil blocked the effects of diazepam on NEOA parameters FLU 2.5 + DZP 2 (*F*_10_ = 18.51, *r* = 0.63, *P* > 0.001) and TTOA FLU 2.5 + DZP 2 (*F*_10_ = 14420, *r* = 1.0, *P* > 0.001). Moreover, flumazenil reversed the effect of the extract at 400 mg/kg on the NEOA parameters FLU 2.5 + EPMG 400 (*F*_10_ = 18.51, *r* = 0.63, *P* > 0.001) and TTOA FLU 2.5 + EPMG 400 (*F*_10_ = 1442, *r* = 1.0, *P* < 0.001), when compared with mice treated only with EPMG 400 mg/kg (Table 5).

### 3.5. Evaluation of Amino Acids Levels

**Figure 5:**
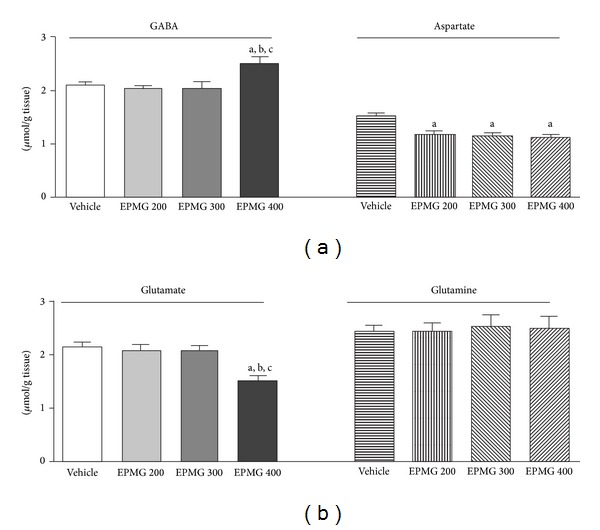
Effect of standardized ethanol extract of leaves of* M. glomerata* on amino acids levels (GABA, aspartate, glutamate, and glutamine) in mice hippocampus. Values represent the mean ± SEM of the number of animals used in experiments. ^a^ *P* < 0.05, compared with negative control, ^b^ *P* < 0.05, compared with EPMG 200 group, ^c^ *P* < 0.05, compared with group EPMG 200, and ^d^ *P* < 0.05, compared with EPMG 300 group (ANOVA and *t*-Student-Newman-Keuls as* post hoc* test).

In this study it was found that the EPMG only at the highest dose increased levels of GABA compared to doses of 200 (*F*_4_ = 5.40, *r* = 0.47, *P* < 0.1) and 300 mg/kg (*F*_4_ = 5.40, *r* = 0.47, *P* < 0.1) and increased the concentration of this amino acid when compared to the negative control (Figure 5).

Moreover, the three tested doses of extract reduced the aspartate concentration (200 mg/kg (*F*_4_ = 6.72, *r* = 0.53, *P* < 0.05), 300 mg/kg (*F*_4_ = 6.72, *r* = 0.53, *P* < 0.05), and 400 mg/kg (*F*_4_ = 6.72, *r* = 0.53, *P* < 0.01)), as compared to negative control. The group treated with the highest dose of EPMG, in turn, produced a decrease in glutamate content as compared to doses of 200 mg/kg (*F*_4_ = 17.20, *r* = 0.74, *P* < 0.001) and 300 mg/kg (*F*_4_ = 17.20, *r* = 0.74, *P* < 0.001) and negative control (*F*_4_ = 17.20, *r* = 0.74, *P* < 0.001) (Figure 5). The doses of 200 mg/kg and 300 mg/kg produced no change in glutamate content when compared with negative control (*P* > 0.05, Figure 5).

The EPMG, in turn, at any test did not produce changes in glutamine levels (*P* > 0.05) when compared with negative control and no change was seen between the doses tested (*P* > 0.05, Figure 5).

## 4. Discussion

Based on those results, it can be verified that standardized ethanol extract of leaves of* M. glomerata* contains the following fatty acids: methyl cinnamate, 2H-1-benzopyran-2-one, (2-hydroxyphenyl)methyl propionate, (Z)-Methyl-hexadec-7-enoate, methyl hexadecanoate, hexadecanoic acid, (Z)-methyl-octadec-9-enoate, octadecanoic acid, and squalene. In other studies with* M. glomerata*, it was shown that this plant has chemical constituents such as coumarin, lupeol, ent-15b-isobutyryloxykaur-16 (17)-en-19-oic acid, sesquiterpenes, diterpenes, kaurenoic acids, cinnamoylgrandifloric acid, stigmasterol, and tannins [26]. Among the chemical constituents cited and identified in this study, coumarin 2H-1-benzopyran-2-one can be considered as a major constituent of* M. glomerata* and therefore can contribute significantly to several of its pharmacological properties [27–29].

The antioxidant study of* M. glomerata* by DPPH^•^ method has been reported by Vicentino and Menezes [30], and antioxidant studies by ABTS^•+^ method in scientific articles were not found. The antioxidant property of a substance is often linked to their ability to donate electron or hydrogen atom to the radical [31]. Thus, the antioxidant capacity of leaves of EPMG can be attributed in part to their chemical composition, found in substances such as cinnamic acid or coumarin derivatives, which have antioxidant capacity [32], and is one of major constituents responsible for the pharmacological properties of* M. glomerata* [33].

In field tests, the administration of EPMG in three doses did not alter the number of groomings, rearings, and crossings, indicating the absence of changes in locomotor activity. The evaluation of the EPMG motor effects in mice in the rotarod test, a model widely used to assess the peripheral motor coordination and neuromuscular blockade, showed that the EPMG (200–300 mg/kg), unlike diazepam (2 mg/kg), had no significant effect on motor coordination. However, with increasing dose of EPMG (400 mg/kg) there were no changes on motor coordination observed by the increased number of falls and decreased length of stay in the rotarod test. It is important to note that despite flocking change in motor coordination, performance in anxiolytic tests (light/dark and EPM tests) was not affected at a dose of 400 mg/kg, as discussed below. Similar result in rotarod test (500 mg/kg) was also found in plants of the same genus as* M. scandens* (L.) Willd. [34] and of different genus as* Passiflora edulis* [35].

The anxiolytic effect of EPMG was evaluated using several models of anxiety in mice. In the light-dark test, the anxiety generated by the conflict between the desire to explore new environments and aversion to an unknown illuminated area can be evaluated according to the residence time in the lighted area, and an increase in this parameter indicates anxiolytic effects [36]. Thus, the results of this study showed that treatment with EPMG increased residence time in the light compartment (Table 3), demonstrating the anxiolytic effect at doses of 200, 300, and 400 mg/kg. The same effect was also seen for diazepam control (Table 3).

In addition to the light-dark test, the test of the elevated plus maze is a widely used experimental model to evaluate the effect of substances on anxiety in animals, and the animals exhibit anxiety behavior when there is usually a reduction in exploiting open arms evidenced by the reduction in the number of entries and time spent in the open arms [37]. Thus, animals treated with antianxiety drugs such as diazepam increase the number of entries and time spent on open arms. Thus, the results of this study indicate that only the 400 mg/kg dose of EPMG reduced the behavioral symptoms of anxiety in the elevated maze test cross, which supports the notion of a dosage selective anxiolytic effect to this test; since the light-dark test, anxiolytic effects were observed at the three doses tested.

In the maze test in high light and dark cross, flumazenil (benzodiazepine receptor antagonist) was used to demonstrate the specific antagonism of GABA_A_ receptors in the anxiolytic action of EPMG mechanism. In this work, the use of EPMG with flumazenil caused a significant decrease in exploration of the open arms of the elevated plus maze, indicating that the anxiolytic effect of EPMG occurs by interaction with GABA_A_ receptors. The same mechanism of anxiolytic action is also attributed to EPMG in light-dark test.

The literature shows that reduction of anxiety is related to elevated GABA levels, with a decrease in content of this amino acid, glutamate, aspartate, and glutamine being directly associated with increased anxiety and behavior [38–40]. Thus, the present study aimed to investigate the effects of amino acids levels (GABA, aspartate, glutamate, and glutamine) on mice hippocampus, in order to correlate it with behavioral studies, investigating the anxiolytic potential and protocols to clarify its possible action mechanism. The results suggest that the anxiolytic effect of this extract may be mediated by decreased glutamate levels (400 mg/kg), as well as by the increased concentration of GABA in mice hippocampus (Figure 5). These outcomes corroborate with studies of Pereira et al. [41] and Galal et al. [42], which demonstrated increased GABA levels and reduced glutamate concentration in mice treated with coumarin and nandrolone decanoate, respectively. As discussed earlier, there are several studies addressing the pharmacological properties of* M. glomerata*; however, this is the first report on its neurochemical effect, whereas other studies focusing on the neuropharmacological properties have been conducted with plants of the same genus, such as* M. scandens* (L.) Willd. [15, 34] and* M. cordata* (Burm.) [43].

## 5. Conclusions 

In conclusion, the present study provides important results with respect to the pharmacological activity of an important medicinal plant. The EPMG demonstrated anxiolytic effects, which may be mediated by GABAergic system, and was able to increase GABA levels and reduce glutamate and aspartate concentrations in mice hippocampus, which can directly and/or indirectly assist in their anxiolytic effect. Although more studies are needed [44], the EPMG could represent an interesting therapeutical strategy in the treatment of anxiety.

## Figures and Tables

**Table 1:** Fatty acids methyl esters and other constituents identified by GC/MS, after hydrolysis and methylation of the standardized ethanol extract of leaves of *M. glomerata* Sprengel.

Compound number	Compound name	R.T.	Area (%)	Main fragments (%)	M^+^/base peak
1	Methyl cinnamate	8.39	2.60	**131** (100); **162** (53); **103** (52); **77 **(37); **51** (30)	162/131
2	2H-1-Benzopyran-2-one	9.49	20.47	**118** (100); **146** (70); **90** (41); **89** (40); **63** (28)	146/118
3	(2-Hydroxyphenyl)methyl propionate	10.65	2.66	**120** (100); **148** (90); **91** (70); **44** (44); **180** (10)	180/120
4	(Z)-Methyl-hexadec-7-enoate	17.49	2.64	**43** (100); **55** (80); **41** (72); **74** (60); **268** (>10)	268/43
5	Methyl hexadecanoate	17.77	36.00	**74** (100); **87** (65); **43** (30); **55** (22); **270** (>10)	270/74
6	Hexadecanoic acid	18.12	5.77	**73** (100); **43** (96); **60** (73); **57** (65); **256** (>10)	256/73
7	(Z)-Methyl-octadec-9-enoate	19.44	15.90	**55** (100); **41** (76); **69** (75); **74** (100); **296** (<10)	296/55
8	Octadecanoic acid	19.63	7.40	**74** (100); **87** (61); **43** (38); **55** (24); **298**	298/74
9	Squalene	26.96	3.89	**69** (100); **81** (69); **41** (28); **95** (20)	—/69

R.T.: retention time; M^+^: ion molecule.

**Table 2:** Evaluation of sedative action mechanism of standardized ethanol extract of leaves of *M. glomerata* in open field test.

Groups (*n*)	Number of crossings	Number of groomings	Number of rearings
Control (12)	98.58 ± 1.19	4.83 ± 0.30	45.33 ± 0.14
DZP 2 (12)	38.58 ± 0.96^a^	2.58 ± 0.15^a^	13.33 ± 0.14^a^
EPMG 200 (12)	94.5 ± 0.15^b^	2.42 ± 0.26	41.0 ± 0.25^b^
EPMG 300 (12)	95.5 ± 0.15^b^	2.58 ± 0.19	46.0 ± 0.25^b^
EPMG 400 (12)	103.5 ± 0.15^b^	2.75 ± 0.18	47.0 ± 0.25^b^
FLU 2.5 (12)	92.5 ± 0.15	4.58 ± 0.31	44.3 ± 0.14
FLU 2.5 + DZP 2 (12)	89.5 ± 0.15^b^	4.67 ± 0.39^b^	45.33 ± 0.14^b^
FLU 2.5 + EPMG 200 (12)	94.5 ± 0.15	2.58 ± 0.29	45.5 ± 0.55
FLU 2.5 + EPMG 300 (12)	97.5 ± 0.15	2.67 ± 0.26	46.67 ± 0.67
FLU 2.5 + EPMG 400 (12)	103.5 ± 0.15	2.75 ± 0.22	47.5 ± 0.78

Values represent the mean ± SEM of number of animals used in experiments. *n* represents the number of animals in each group. ^a^ *P* < 0.05, compared with negative control (vehicle), ^b^ *P* < 0.05, compared with diazepam group (ANOVA and *t-*Student-Newman-Keuls as *post hoc* test). EPMG: standardized ethanol extract of leaves of *M. glomerata*; DZP: diazepam; and FLU: flumazenil.

**Table 3:** Evaluation of anxiolytic action mechanism of standardized ethanol extract of leaves from *M. glomerata* in light dark box test.

Groups (*n*)	Residence time in the light compartment (seconds)
Control (12)	104.3 ± 0.33
DZP 2 (12)	158.3 ± 0.48^a^
EPMG 200 (12)	133.3 ± 0.38^a^
EPMG 300 (12)	157.3 ± 0.41^a^
EPMG 400 (12)	169.3 ± 0.38^a,b,c,d^
FLU 2.5 (12)	107.3 ± 0.38
FLU 2.5 + DZP 2 (12)	102.7 ± 0.38^b^
FLU 2.5 + EPMG 200 (12)	102.3 ± 0.38
FLU 2.5 + EPMG300 (12)	104.7 ± 0.51
FLU 2.5 + EPMG400 (12)	108.7 ± 0.62^e^

Values represent the mean ± SEM of the number of animals used in experiments. *n* represents the number of animals in each group. ^a^ *P* < 0.05 compared with vehicle (negative control), ^b^ *P* < 0.05, compared with diazepam group, ^c^ *P* < 0.05, compared with group EPMG 200, ^d^ *P* < 0.05 compared with EPMG 300 group, ^e^ *P* < 0.05, compared with group EPMG 400 (ANOVA and *t*-Student-Newman-Keuls as *post hoc* test). EPMG: standardized ethanol extract of leaves of *M. glomerata*; DZP: diazepam; and FLU: flumazenil.

**Table 4:** Evaluation of anxiolytic action mechanism of standardized ethanol extract of leaves of *M. glomerata* on motor coordination in rotarod test.

Groups (*n*)	Number of falls	Residence time on the rotating bar (seconds)
Control (12)	1.67 ± 0.14	176.1 ± 1.02
DZP 2 (12)	2.83 ± 0.17^a^	167.4 ± 3.18^a^
EPMG 200 (12)	1.33 ± 0.31	177.3 ± 0.81
EPMG 300 (12)	1.50 ± 0.19	176.3 ± 0.60
EPMG 400 (12)	2.08 ± 0.47^a^	174.1 ± 1.29^a^
FLU 2.5 (12)	1.58 ± 0.19	177.5 ± 0.36^b^
FLU 2.5 + DZP 2 (12)	1.67 ± 0.31^b^	176.5 ± 0.36
FLU 2.5 + EPMG 200 (12)	1.33 ± 0.33	177.8 ± 0.27
FLU 2.5 + EPMG300 (12)	1.58 ± 0.23	177.4 ± 0.47
FLU 2.5 + EPMG400 (12)	1.58 ± 0.26^c^	177.7 ± 0.79^c^

Values represent the mean ± SEM of the number of animals used in experiments. *n* represents the number of animals in each group. ^a^ *P* < 0.05 compared with negative control (vehicle), ^b^ *P* < 0.05, compared with diazepam group, and ^c^ *P* < 0.05, compared with FLU 2.5 + EPMG 400 group (ANOVA and *t-*Student-Newman-Keuls as *post hoc* test). EPMG: standardized ethanol extract of leaves of *M. glomerata*; DZP: diazepam; and FLU: flumazenil.

**Table 5:** Evaluation of anxiolytic action mechanism of standardized ethanol extract of leaves from *M. glomerata* in elevated plus-maze test.

Groups (*n*)	NEOA	TTOA (seconds)
Control (12)	11.67 ± 0.5	123.8 ± 0.41
DZP 2 (12)	15.83 ± 0.24^a^	207.3 ± 0.70^a^
EPMG 200 (12)	11.33 ± 0.38	123.6 ± 0.40
EPMG 300 (12)	11.67 ± 0.75	124.6 ± 0.40
EPMG 400 (12)	17.08 ± 0.78^a,b,c,d^	226.3 ± 0.62^a,b,c,d^
FLU 2.5 (12)	10.92 ± 0.68	123.5 ± 0.56
FLU 2.5 + DZP 2 (12)	10.58 ± 0.48^b^	124.6 ± 0.40^b^
FLU 2.5 + EPMG 200 (12)	10.25 ± 0.46	122.8 ± 0.28
FLU 2.5 + EPMG 300 (12)	10.58 ± 0.48	123.6 ± 0.40
FLU 2.5 + EPMG 400 (12)	11.5 ± 0.47^e^	123.8 ± 0.55^e^

Values represent the mean ± SEM of the number of animals used in experiments. *n* represents the number of animals in each group. ^a^ *P* < 0.05 compared with negative control, ^b^ *P* < 0.05, compared with diazepam group, ^c^ *P* < 0.05, compared with group EPMG 200, ^d^ *P* < 0.05 compared with EPMG 300 group, and ^e^ *P* < 0.05, compared with FLU 2.5 + EPMG 400 group (ANOVA and *t-*Student-Newman-Keuls as *post hoc* test). EPMG: standardized ethanol extract of leaves of *M. glomerata*; DZP: diazepam; and FLU: flumazenil.
